# Metabolic Interplay between Tumour Cells and Cancer-Associated Fibroblasts (CAFs) under Hypoxia versus Normoxia

**DOI:** 10.21315/mjms2018.25.3.2

**Published:** 2018-06-28

**Authors:** Septelia Inawati Wanandi, Sri Suciati Ningsih, Hijrah Asikin, Rendy Hosea, Gladies Mercya Grameinie Neolaka

**Affiliations:** 1 Department of Biochemistry and Molecular Biology, Faculty of Medicine, Universitas Indonesia, Jalan Salemba Raya No. 6, Jakarta 10430, Indonesia; 2 Master Program in Biomedical Sciences, Faculty of Medicine, Universitas Indonesia, Jalan Salemba Raya No. 4, Jakarta 10430, Indonesia

**Keywords:** cancer-associated fibroblasts, hypoxia, lactate dehydrogenases, monocarboxylate transporters, Warburg effect, reverse Warburg effect

## Abstract

The growth of tumour cells is closely related to cancer-associated fibroblasts (CAFs) present within their microenvironment. CAFs, the most abundant cells in tumour stroma, secrete growth factors that play pivotal roles in tumour cell proliferation, metabolism, angiogenesis and metastasis. Tumour cells adapt to rapid environmental changes from normoxia to hypoxia through metabolic interplay with CAFs. In this mini review, we discuss the role of lactate dehydrogenases (LDHs) and monocarboxylate transporters (MCTs) on the metabolic interplay between tumour cells and CAFs under hypoxia compared to normoxia. The LDHs catalyse the interchange of lactate and pyruvate, whereas MCTs facilitate the influx and efflux of monocarboxylates, especially lactate and pyruvate. To sum up, tumour cells switch their metabolic state between glycolysis and oxidative phosphorylation through metabolic interplay with CAFs, which exhibit the Warburg effect under hypoxia and reverse Warburg effect under normoxia.

## Introduction

According to the World Health Organization (WHO), cancer is the uncontrolled growth of cells which can invade healthy tissue and spread to distant sites in the body (1). Other terms used for cancer include malignant tumour and neoplasm. Cancer cells ignore healthy cellular growth and death signals, and thus these cells can proliferate in an uninhibited and unlimited manner. Similar to normal cells, the growth of cancer cells is closely related to their microenvironment or local surroundings, including stroma and the extracellular matrix in which the cancer cells exist. The cancerous cells interact with their microenvironment through various chemical and physical signals that contribute to cancer cell growth and death. As such, stroma supports tumour growth by secreting growth factors for proliferation and metastasis of cancer cells. Furthermore, the microenvironment conditions cancer cells to allow them to survive in extreme conditions, such as acidosis and hypoxia (2, 3).

Stroma consists of indistinguishable cells. Stromal cells show distinct morphology and varying degrees of differentiation and invasiveness. Some cell populations, such as fibroblasts, adipocytes, endothelial cells and inflammatory cells, are embedded in a specific extracellular matrix. In cancer stroma, normal fibroblasts have been transformed into cancer-associated fibroblasts (CAFs), which are characterised by the presence of several markers such as alpha-smooth muscle actin (α-SMA), platelet-derived growth factor-β receptor (PDGFR-β) and vimentin (4, 5). CAFs secrete factors that play crucial roles in cancer cell proliferation, metabolism, angiogenesis and metastasis. Cancer cells and CAFs communicate with each other in many ways, including through metabolic interplay in hypoxic conditions. CAFs may also undergo an aerobic glycolysis cycle that produces high-energy metabolites, which can be exported and taken up by tumour cells to produce high amounts of energy through oxidative phosphorylation (3, 4).

In general, cell metabolism follows the fundamental principle of harvesting energy from catabolism of biomacromolecules, such as carbohydrates, proteins and lipids, and synthesising molecules using the energy yield. As the solid tumour grows larger, it rapidly outgrows its blood supply, leading to a concentration of oxygen in tumour parts that is relatively lower than the oxygen concentration in healthy tissues, which is known as tumour hypoxia. In order for malignant cells to survive in hypoxic conditions, they adapt by switching their metabolic programme. In tumour cells (glycolytic cells), glucose is converted into lactate even though there is adequate oxygen in the microenvironment. This process is known as the Warburg effect (6). Lactate dehydrogenases (LDHs) are metabolically important enzymes involved in the critical step of inter-conversion of lactate into pyruvate in tumour cells. Several studies have indicated that LDH expression and activity could be used as a hallmark to determine metabolic state of cancer cells (7, 8). The excess lactate produced by glycolytic tumour cells is removed from the tumour microenvironment through uptake by CAFs, which act as oxidative cells. The lactate that is taken up by CAFs is used as fuel by incorporating it into oxidative phosphorylation in the mitochondria. In contrast to the Warburg effect, hypoxic CAFs (glycolytic cells) in the microenvironment can export lactate into tumour cells (oxidative cells), which will then use the lactate to undergo oxidative phosphorylation. This phenomenon is called the reverse Warburg effect (9). Lactate transport between tumour cells and CAFs is mediated by MCTs (monocarboxylate transporters), primarily MCT1, MCT2 and MCT4. The activity of MCTs is concomitant with the activity of LDHs, which converts pyruvate into lactate in the last step of anaerobic glycolysis (7, 10).

An understanding of the metabolic interplay between solid tumour and stromal cells may aid in the eradication of cancer through a tumour microenvironment approach (11–13). In this review, we discuss the communication between CAFs and tumour cells which influences the metabolic switch in both cells. Along with the metabolic interplay between CAFs and tumour cells, the role of MCT1 and MCT4 on lactate transport between cells is also discussed.

## Glycolysis under Tumour Hypoxia

Eukaryotic cells have a highly organised structure and require energy to maintain their biological functions for survival. This energy can be acquired from chemical reactions involving the breaking of chemical bonds, predominantly glucose chemical bonds. Glycolysis is a major pathway in glucose metabolism, which, uniquely, can occur in both aerobic and anaerobic conditions. Glycolysis has two main functions: breaking down glucose to generate ATP (catabolism) and providing building blocks for biosynthesis. Glycolysis consists of multistep reactions catalysed by hexokinase, phosphofructokinase and pyruvate kinase. Each enzyme catalyses a different chemical reaction in the glycolytic pathway, thus serving as control sites for the total rate of glycolysis (14).

**Figure 1:**
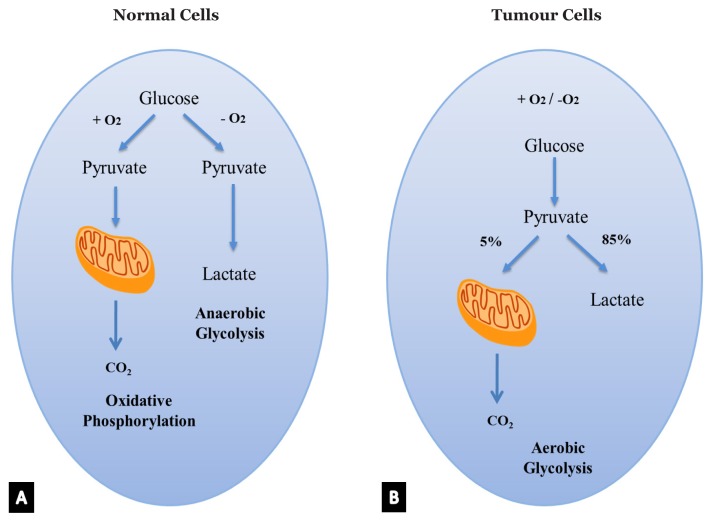
Schematic representation of the differences between oxidative phosphorylation. A. Anaerobic glycolysis in normal cells. B. Anaerobic glycolysis in tumour cells (Warburg effect)

Which pathway a cell will undergo in glucose metabolism depends upon the availability of oxygen and NAD^+^, which is converted into NADH, the electron carrier molecule (Figure 1). To maintain glycolysis in normal differentiated cells, NADH must be converted back to its oxidised form, NAD^+^. As shown in Figure 1A, when a sufficient amount of oxygen is available, pyruvate will be converted into acetyl CoA, which further enters the Krebs cycle (TCA cycle) and oxidative phosphorylation to be fully oxidised to CO_2_ in the mitochondrial matrix. NADH generated by the Krebs cycle is fed into the oxidative phosphorylation pathway and electron transfer from NADH to O_2_ regenerates NAD^+^. Oxidative phosphorylation (OXPHOS) produces about 32 to 38 ATPs per molecule of glucose. When oxygen is limited (anaerobic glycolysis), for example, in highly active skeletal muscles, NAD^+^ regeneration is achieved by the reduction of pyruvate to lactate, as pyruvate cannot be transferred into the mitochondrial respiratory chain. This allows anaerobic glycolysis to continue; nevertheless, this pathway generates significantly less ATP, only two molecules per molecule of glucose oxidised (14).

Rapidly proliferating cells in solid tumour consume a significant amount of oxygen, thereby limiting the availability of oxygen supplied by local blood vascularisation. This condition results in a relatively hypoxic microenvironment, also known as tumour hypoxia. As a consequence, tumour cells switch their metabolic programme from that of oxidative cells to that of glycolytic cells (6). Changes in the metabolic programme within tumour cells were first proposed by the 1920 Nobel Prize winner, Otto Warburg (15). Warburg (15) found that even in the presence of adequate oxygen, tumour cells prefer to convert most glucose (85%) to lactate. This seems like a paradox, considering that oxidative phosphorylation yields ATP more efficiently. However, this phenomenon could be explained if we consider the fact that relative tumour hypoxia occurs even when adequate amounts of oxygen are present. A similar pathway can also be observed in other types of rapidly proliferating cells, such as in fibrosis and keloids. Therefore, this pathway is known as aerobic glycolysis or the Warburg effect (Figure 1B). Because the mitochondria remain functional, oxidative phosphorylation continue at a low rate (5%) in normal proliferating cells as well as in tumour cells. Notably, a mechanism that solves the paradox is increasing glucose uptake to compensate for the low efficiency of ATP production in tumour cells. This could be resolved by upregulating GLUT, a glucose transporter. This particular characteristic has been taken advantage of in clinical applications in detecting tumours with fluorodeoxyglucose positron emission tomography (FDG-PET) (11).

A solid tumour has a heterogeneous distribution of oxygen, rendering some parts of it hypoxic. Hypoxic tumour cells located far from blood vessels are prone to using glucose to produce energy and lactate. This metabolite is exported as a main substrate for OXPHOS to the oxidative stromal cells near blood vessels. This relationship describes the mutually symbiotic metabolism between glycolytic tumour cells and oxidative cells in the microenvironment which could maintain the proliferation of tumour cells under hypoxic conditions (16).

Malignant cells must be able to survive under hypoxia, and therefore, a mechanism to adapt to hypoxia must be developed. Normal cells and tumour cells have a similar adaptation mechanism which includes the stabilisation of hypoxia-inducible factor (HIF)-1α (6). HIF1-α is hydroxylated under normoxia conditions and is recognised by VHL (Von Hipple Lindau) proteins as degraded. At low oxygen levels, HIF-1α protein accumulates and binds to HIF-1β, forming HIF-1, the active transcription factor which further activates the transcription of several target genes in response to hypoxia. Genes targeted by HIF-1 express growth factors that support angiogenesis, glucose transporters and enzymes that function in glycolysis, such as LDH. Target genes regulated by HIF-1a are expressed in many types of cancer and it has been suggested to be correlated with poor prognosis. Overall, the transcription activation by HIF-1α enhances cellular glucose uptake and glycolysis (17).

## Cancer-Associated Fibroblasts (CAFs)

Stromal cells are the most essential components in the tumour microenvironment. Stromal cells are not tumourigenic but support the growth and development of tumour cells. Several types of stromal cells in the tumour microenvironment, including endothelial cells, pericyte cells, immune cells, tumour-associated macrophages (TAMs), mesenchymal stem cells (MSCs) and CAFs, cooperate together in the proliferation and metastasis of tumours (4, 18).

MSCs are multipotent cells with the ability to differentiate into osteoblasts, chondrocytes and adipocytes. These cells are derived from bone marrow or adult-derived stem cells and have the ability to self-renew. MSCs support the growth and development of the tumour by assisting tumour immune evasion and by acting as an immunosuppressant together with regulatory T cells and dendritic cells. Moreover, MSCs release growth factors into the tumour microenvironment at the primary site of the tumour. As the tumour grows, MSCs are recruited to the growing tumour in response to chemokines secreted by the tumour cells and then induced to differentiate into CAFs and to acquire pro-tumour characteristics (3, 19).

CAFs are the most abundant cells in tumour stroma. Instead of supporting tumour growth, CAFs may promote angiogenesis and epigenetic alteration, as well as inducing epithelial-mesenchymal transition (EMT). Activation of the EMT programme mediates the metastasis of cancer cells originating from epithelial cells. CAFs express several mesenchymal-specific proteins, such as fibroblast-specific protein (FSP-1), fibroblast-activating protein-alpha (FAP-a), vimentin and alpha-smooth muscle actin (α-SMA), the prototypical marker for myofibroblasts, differentiating these cells from normal fibroblasts. These intracellular and plasma membrane–associated proteins have been used as markers to detect the presence of CAFs in tumours. As the tumour grows, some precursor cells, such as normal fibroblasts, bone marrow mesenchymal cells (BMCs) and MSCs, might be transformed into CAFs and acquire pro-tumour properties. Activation of CAFs from precursor cells might be induced by transforming growth factor-β (TGF-β) secreted by tumour cells (5, 20). The dominant growth factor responsible for differentiation of CAFs remains to be elucidated.

As mentioned above, CAFs are not tumourigenic, but they support the growth and metastasis of tumour cells. This function is mediated through paracrine signalling, which includes secretion of several growth factors such as hepatocyte growth factor (HGF), epidermal growth factor (EGF), insulin-like growth factor (IGF), nerve growth factor (NGF), fibroblast growth factor (FGF), granulocyte colony stimulating factors (G-CSF), TGF-β and Wnt. In addition, CAFs secrete several chemokines such as interleukin, CXCL12, CXCL14, CCL7 and vascular endothelial growth factor (VEGF) to induce tumour angiogenesis, which is an important step in tumourigenesis. Previous studies have illustrated that the cell–cell communication between CAFs and tumour cells involves the production of chemokines such as interleukin 6 (IL6) which alter the tumour microenvironment by recruiting macrophages and increasing the ability of CAFs to produce VEGF, triggering tumour angiogenesis (19, 21).

CAFs also display other pro-tumouric activity that is not associated with cell–cell signalling. CAFs secrete extracellular matrix components which increase the rigidity of solid tumours. Connective tissue and extracellular matrix components such as collagen, laminin, fibronectin and some proteoglycans provide mechanical support for cells, facilitate communication between cells, and therefore provide substrates for cell migration. Besides releasing chemokines, growth factors and matrix components, CAFs have another crucial function: supporting tumour growth and metastasis (20, 22). This is achieved by providing an alternative carbon source to oxidative tumour cells. Additional metabolic aspects are elaborated in the next section.

## Lactate and Lactate Dehydrogenase (LDH)

Lactate (2-hydroxypropionate) is the anion produced from the dissociation of lactic acid, which is the end product of anaerobic glycolysis. In nature, lactate exists in two stereoisomeric forms due to the presence of an asymmetric carbon atom, namely L-lactate and D-lactate. Both stereoisomers of lactate are generated from and metabolised to pyruvate by the action of the lactate dehydrogenase (LDH) enzyme, which is isomer-specific. The enzyme for the production and metabolism of L-lactate is L-LDH and for D-lactate is D-LDH. However, mammalian cells contain only L-LDH, and therefore, the lactate produced in humans is almost exclusively L-lactate (23).

In general, LDH (EC 1.1.1.27) is an enzyme regulated by HIF-1a that catalyses the NADH-dependent reduction of pyruvate to lactate or NAD^+^-dependent oxidation of lactate to pyruvate (24). LDH has two different subunits, the M subunit and the H subunit. The two LDH subunits combine to form five LDH isozymes, LDH1 (H_4_), LDH2 (H_3_M_1_), LDH3 (H_2_M_2_), LDH4 (H_1_M_3_) and LDH5 (M_4_), which are distributed in distinct organs (25). LDH-A is encoded by the LDH-A gene, which is located on chromosome 11p15.4, whereas LDH-B is encoded by the LDH-B gene on chromosome 12p12.2-p12.1. Although these five isozymes have the same enzyme activity, they show distinct tissue distribution, suggesting functional variation. The isozyme in aerobic organs mostly consists of the LDH-B subunit, while the isozyme in anaerobic organs is primarily LDH-A (23, 24, 26).

LDH-A and LDH-B have the same active site and the same amino acids participating in the reaction; nevertheless, they exhibit a notable difference with regard to one amino acid which makes up the tertiary structure of LDH: alanine (in LDH-A) and glutamine (in LDH-B). This tiny change is believed to be essential to the differences in the kinetics and activity between these subunits. LDH-A catalyses the reduction of pyruvate to lactate using NADH and H^+^, whereas LDH-B catalyses the oxidation of lactate to form pyruvate using NAD^+^ (23, 27).

## Monocarboxylate Transporters (MCTs)

Lactate and pyruvate are monocarboxylates that play important roles in cellular metabolism and metabolic communication between tissues. The cytoplasmic level of lactate and pyruvate is regulated by monocarboxylate transporters (MCTs), transmembrane proteins that mediate proton-linked transport of monocarboxylate molecules including lactate, pyruvate and ketone bodies across cell membranes (28).

MCTs have 12 transmembrane (TM) helices with intracellular C- and N-termini and a large intracellular loop between TM 6 and 7. There are 14 subtypes of MCTs which are encoded by the SLC16A gene family. MCT1, 2, 3 and 4 drive essential metabolic roles in almost every tissue. These four isozymes can be differentiated from each other, because they have distinct properties, expression profiles, and subcellular localisation corresponding to the particular metabolic needs of the tissue in which they are present (28). Recent study has revealed the physiological and pathological roles of MCT1–4 expression in most types of cancer (29, 30).

To meet the high energy requirements of tumour cells, lactate is used as an alternative carbon source for OXPHOS. MCT1 facilitates the influx of lactate into oxidative cells. Afterwards, the intracellular lactate is converted by LDH-B into pyruvate, which is used as an energy source for oxidative phosphorylation. The upregulation of MCT1 expression is associated with an increase of citrate synthase activity. MCT4 is a lactate efflux facilitator that transports lactate from glycolytic cells. Hence, the expression of MCT1 is upregulated in highly oxidative cells as a response to the high activity and energy demand of those cells, whereas the expression of MCT4 is upregulated in high glycolytic cells as a response to hypoxia (29–31).

MCT1 and MCT4 are known to have important roles in the metabolic interplay between tumour cells and stromal cells in the tumour microenvironment. The proteins are distinctly expressed in glycolytic and oxidative cells and therefore could be used as functional biomarkers of symbiotic metabolism in some types of cancer such as those of the head and neck (HNSCC) (30, 32). The functions of both proteins depend on their interaction with the chaperone protein CD147, which is required for the trafficking process and cellular localisation of MCT1 and MCT4 at the post-translational level (31).

## Metabolic Interplay between Tumour Cells and CAFs

According to the Warburg effect, tumour cells produce a large amount of lactate as a final product of glycolysis. High glycolytic tumour cells must thus establish an efficient way to remove excess intracellular lactate. Obviously, an over-accumulation of lactate in tumour cells will be imported by CAFs, which use lactate for their own energy needs (6).

**Figure 2:**
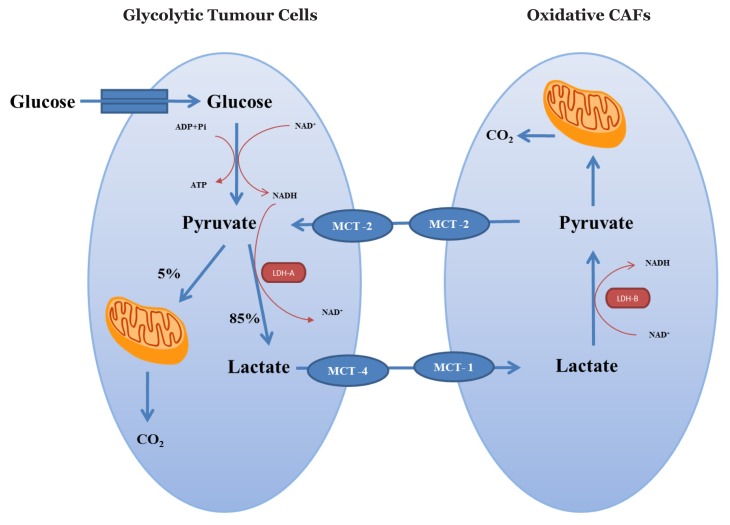
Metabolic interplay between glycolytic tumour cells and oxidative CAFs in hypoxia (Warburg effect)

The metabolic interplay between tumour cells and CAFs involves MCTs, which facilitate the export and import of lactate between both cells. The ability of CAFs to use lactate as a carbon source is supported by the high expression of MCT1 as an influx facilitator of lactate in CAFs (7). Excessive amounts of pyruvate in CAFs can also be exported by MCT2 to be used as an energy source for tumour cells (Figure 2). The import–export of pyruvate in tumour cells is facilitated by MCT2 (33). Hence, oxidative tumour cells can obtain additional pyruvate to produce energy by OXPHOS through the ability of CAFs to recycle lactate.

**Figure 3:**
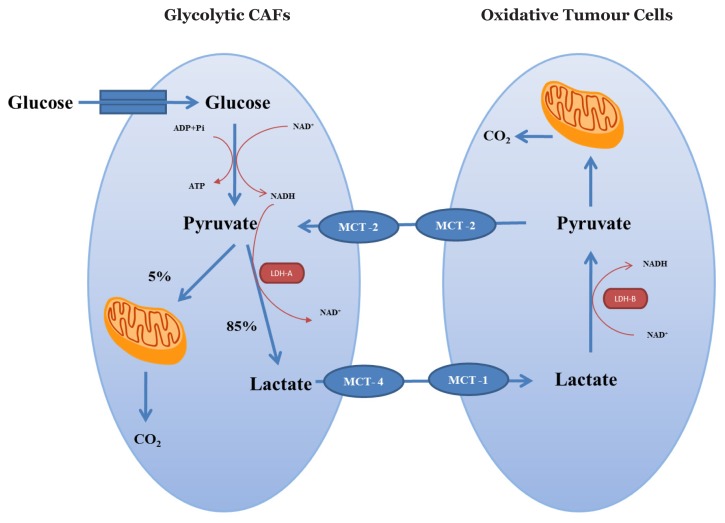
Metabolic interplay between oxidative tumour cells and glycolytic CAFs in normoxia (Reverse Warburg effect)

Interestingly, CAFs are also able to undergo aerobic glycolysis, similar to tumour cells. In some types of tumours, CAFs switch their metabolism from oxidative phosphorylation to glycolysis. This event is known as the reverse Warburg effect (Figure 3). Zhang et al. (34) demonstrated that isocitrate dehydrogenase-3α (IDH3α) suppression led to prolyl hydroxylase domain-2 (PHD2) inhibition and HIF-1α protein stabilisation. The accumulation of HIF-1α reprogrammed the metabolic state of CAFs into glycolysis by up-regulating the expression of genes for glycolytic enzymes, increasing glucose uptake and inhibiting OXPHOS (34).

The ability of CAFs to undergo glycolysis and produce lactate has been demonstrated using breast epithelial tumour cells secreting H_2_O_2_ (35). Secreted H_2_O_2_ elicited an oxidative stress response in stromal cells and increased aerobic glycolysis enzymes, such as LDH-A (36). The upregulation of MCT4 expression in CAFs, as demonstrated using immunostaining assays, indicates the reverse Warburg effect. MCT4 is used for removal of excess lactate from the glycolytic CAFs. That study showed that stromal CAFs are prone to switching their metabolic programme to aerobic glycolysis (35). Furthermore, the crosstalk between cancer cells and CAFs has been demonstrated through a co-culture study. When CAFs were co-cultured with breast tumour cells, they could increase the gene expressions to upregulate carbohydrate metabolism, including glycolysis, glycogenesis and glucose transport. In contrast, the breast cancer cells co-cultured with fibroblasts upregulated genes related to mitotic response and the TCA pathway. An interesting result from the co-culture study is that most of the upregulated genes in cancer cells are found to be downregulated in CAFs, and vice versa (37). The reciprocal nature of gene expressions in cancer cells and CAFs reveals that there is a metabolic interplay between both cells.

Under normoxia, tumour cells become more oxidative and induce CAFs to undergo aerobic glycolysis, resulting in an increase in lactate production in CAFs. Lactate could be used as a significant energy source for tumour cells when it is converted to pyruvate and proceeds through oxidative phosphorylation (Figure 3). In addition, high-energy metabolites such as L-lactate and 3-hydroxy-butyrate (ketone bodies) could stimulate tumour growth and act as chemoattractants that stimulate metastasis (38).

By undergoing aerobic glycolysis to produce lactate, CAFs have the same metabolism as tumour cells. However, the aerobic glycolysis in CAFs is slightly different from that in tumour cells in terms of cell proliferation. The proliferation capability of CAFs is lower than that in tumour cells because of CAF’s lack of ability to use the metabolites of aerobic glycolysis for biosynthesis. Consequently, these metabolites are secreted to the adjacent tumour cells, which then consume them and use them as biosynthesis factors to support tumour growth and metastasis.

## Summary

Tumour cells adapt well to rapid changes in oxygen availability, from normoxic to hypoxic conditions, through metabolic interplay with stromal cells. CAFs in the stroma produce growth factors, chemokines, extracellular matrix and metabolites that can be used by tumour cells. Tumour cells survive by establishing this metabolic interplay with CAFs, switching their metabolic state between glycolysis and oxidative phosphorylation. As a result, tumour cells exhibit the Warburg effect under hypoxic conditions and the reverse Warburg effect under normoxic conditions. The metabolic interplay between tumour cells and CAFs supports the proliferation and metastasis of tumour cells. The metabolic state of tumour cells can be determined by the ratio of LDH-B (dominant in aerobic metabolism) to LDH-A (dominant in anaerobic metabolism) expression, which is essential for the interchange between lactate and pyruvate. The export and import of lactate between tumour cells and CAFs is facilitated by MCT1 and MCT4 transporters, which could be used as functional biomarkers to determine the symbiotic mechanisms between oxidative and glycolytic cells and between tumour cells and CAFs in tumour stroma. Inhibition of MCT1 and MCT4 may disrupt the metabolic interplay between tumour cells and CAFs, suggesting that these proteins are potential targets for anticancer therapeutics.
